# Higher Unilateral Muscle Imbalance at the Contralateral Knee 6 Months after Anterior Cruciate Ligament Reconstruction

**DOI:** 10.3390/sports12090243

**Published:** 2024-09-04

**Authors:** Leonor López de Dicastillo, Jesús Villalabeitia, Diego Delgado, Cristina Jorquera, Renato Andrade, João Espregueira-Mendes, Patrick Middleton, Mikel Sánchez

**Affiliations:** 1 Arthroscopic Surgery Unit, Hospital Vithas Vitoria, 01008 Vitoria-Gasteiz, Spain; leonor.lopez@ucatrauma.com; 2 Advanced Physiotherapy Unit, Hospital Vithas Vitoria, 01008 Vitoria-Gasteiz, Spain; jesus.villalabeitia@ucatrauma.com (J.V.); p.middleton40@icloud.com (P.M.); 3 Advanced Biological Therapy Unit, Hospital Vithas Vitoria, 01008 Vitoria-Gasteiz, Spain; diego.delgado@ucatrauma.com (D.D.); cristina.jorquera@ucatrauma.com (C.J.); 4 Clínica Espregueira-FIFA Medical Centre of Excellence, 4350-415 Porto, Portugal; randrade@espregueira.com (R.A.); jem@espregueira.com (J.E.-M.); 5 Dom Henrique Research Centre, 4350-415 Porto, Portugal; 6 Porto Biomechanics Laboratory (LABIOMEP), Faculty of Sports, University of Porto, 4200-450 Porto, Portugal; 7 School of Medicine, University of Minho, 4710-057 Braga, Portugal; 8 ICVS/3B’s—PT Government Associate Laboratory, 4710-057 Braga/Guimarães, Portugal; 9 3B’s Research Group—Biomaterials, Biodegradables and Biomimetics, University of Minho, Headquarters of the European Institute of Excellence on Tissue Engineering and Regenerative Medicine, 4806-909 Barco, Portugal

**Keywords:** anterior cruciate ligament, hamstrings, quadriceps, isokinetic

## Abstract

There are a considerable number of patients who, after anterior cruciate ligament reconstruction (ACL), suffer from relapses or reduced performance. Data collected from isokinetic dynamometry can provide useful information on the condition of the knee during rehabilitation. Seventy-one young sports patients with ACL reconstruction performed concentric (CON) isokinetic dynamometry (CON/CON 90°/s and CON/CON 240°/s) to assess the muscle strength of the quadriceps (Q) and hamstrings (H) in both knees at 6 months after ACL reconstruction. Limb symmetry index (LSI) and the H/Q ratio were calculated. Comparative statistical tests and multivariate regression were performed. At 90°/s, 57 patients (80.3%) had an LSI below 90% for quadriceps and 28 (60.6%) for hamstring. The number of imbalanced patients according to H/Q ratio was higher in the non-operated knee (*n* = 56, 78.9%) (*p* < 0.001). At 240°/s, 49 cases (69.1%) had LSI values above 90% for quadriceps and 37 (52.1%) for hamstrings. Regarding H/Q, imbalanced cases were higher in the non-operated limb (*n* = 60, 84.5%) (*p* < 0.001). Strength data at 6 months after ACL reconstruction and post-operative rehabilitation indicated greater unilateral (H/Q) muscle imbalance in the non-operated knee than in the operated knee. Most patients did not achieve the adequate LSI values.

## 1. Introduction

Anterior cruciate ligament (ACL) tears are a common injury not only affecting professional athletes but also the general population, whose annual incidence is 68.6 per 100,000 persons. It has a higher incidence in men than in women, with a peak incidence between the ages of 15 and 25 [1].

The high incidence of ACL tears is also accompanied by an increasing number of ACL reconstruction [2,3,4,5]. For professional athletes, ACL reconstruction is key not only for returning to sports but also to achieve the same level of performance as before the injury [6]. Unfortunately, despite the fact that ACL reconstruction is the gold-standard treatment, there is still a considerable proportion of individuals suffering worsening performance after ACL rupture [7,8], which can be conditioned by several factors related to the patient, surgery, or rehabilitation [9].

Returning to sports after ACL reconstruction is not without risk of further knee injuries, with around 23% of young athletes suffering a secondary ACL injury after they returned to sports [10]. According to several studies, athletes may return to sports as early as six months after surgical repair [11,12,13,14,15]. Before, athletes should be tested to achieve adequate information and to make the right decision. An early return to sports or inadequate recovery of the knee function may lead to subsequent ACL re-tears [16]. Measurement of knee muscle strength is one of the strongest indicators of knee function that is associated with the risk of secondary ACL injuries [16,17] and often used as a criterion to decide if the athlete is ready to return to sports [18]. The isokinetic strength evaluation can be compared to the non-operated knee to calculate the bilateral muscle strength imbalance and calculate the limb symmetry index (LSI) [19], with an LSI value above 90% being considered an appropriate outcome of ACL reconstruction [20]. The hamstring to quadriceps (H/Q) ratio, which relates the peak torque of the hamstrings to those of the quadriceps, is also an important strength indicator of unilateral muscle balance. It is a good indicator of knee unilateral muscle balance, which could provide valuable information for the rehabilitation of ACL reconstruction [21,22]. In addition to the rehabilitation process, the success of ACL reconstruction can be conditioned by different variables related to the patient or the surgical intervention, as shown by numerous studies [10,23,24,25,26,27,28]. However, studies analyzing the relationship of this type of variable with isokinetic data are scarce, and they do not usually include factors such as associated injuries or certain surgical procedures [29,30,31].

The aim of the present study was to analyze the unilateral and bilateral knee muscle strength balance in patients with ACL reconstruction, as well as the prognostic factors that may affect the isokinetic muscle strength at 6 months after ACL reconstruction.

## 2. Materials and Methods

### 2.1. Patient Enrollment

The study was designed as a prospective observational study. Participants were enrolled consecutively between 2020 and 2023 at the same medical center (Advanced Physiotherapy Unit, Arthroscopic Surgery Unit, Vitoria-Gasteiz, Spain). Ethical approval (protocol no: EPA2016067) was obtained from the Ethics Committee of the Basque Country (February 2017), and informed consent was obtained from all patients. The study was carried out in accordance with the International Declaration of Helsinki (Fortaleza, Brazil; 2013) and Good Clinical Practice.

Inclusion criteria for eligible patients were reviewed by the same physician (L.L.). They were older than 18 years of age of both sexes and active in sports. They were required to have undergone ACL reconstruction at our center and to have signed the study consent form. Patients who did not perform isokinetic dynamometry to assess the knee at 6 months after intervention were excluded from analysis.

A power analysis was conducted to estimate the minimum sample size needed to achieve 90% power at a 5% level of significance for the primary outcome measures. The minimum difference to be detected is 25 N-m of maximum isometric contraction force with a standard deviation of 45 points according to preliminary data. This analysis suggested a minimum of 69 patients.

### 2.2. Baseline Evaluation

All patients were evaluated at baseline to collect patient-related data, including age, sex, and professional sports practice. The collected variables related to surgery were the type of ACL surgery (primary, revision), graft origin (autograft, allograft), augmentation with anterolateral reconstruction, and meniscal repair during the surgery.

### 2.3. Surgical Technique

The patient is positioned supine and anesthesia, prophylactic antibiotic treatment, and saline are administered. The joint is assessed by arthroscopy to detect any associated knee injuries such as meniscal, synovial, or chondral damage.

Condyloplasty is performed as soon as the ACL remnants are cleaned to prevent future graft impingements, especially in chronic cases with a narrower groove. This surgical step is also important to promote the correct location of the femoral tunnel placement of the graft. A bed of bleeding cancellous bone is created, providing cells and proteins that will enhance the integration of the graft. Once the joint site is prepared, the autologous grafts are obtained (hamstring tendons). If allografts are used, they are prepared beforehand.

The tunnels are produced using the selected procedures and guides. Fixation of the graft to the femur is performed using the TightRope cortical implant (Arthrex, Naples, FL, USA). Tibial fixation is performed by means of bone plugs removed during bone tunneling. The bone grafts are reimplanted after graft positioning, and the tibial tunnel is sealed, providing biological fixation.

Platelet-rich plasma was used as biological augmentation at the different steps, including the infiltration and soaking of the grafts and bone plugs, the intraosseous infiltration of the tunnels and surroundings, and intra-articular injection as a final step.

### 2.4. Post-Operative Protocol and Recommendations

During the first 14 days following surgery, patients were instructed to keep relative rest with the knee extended and apply ice on a regular basis to reduce inflammation and pain. Rehabilitation was initiated in a progressive and pain-free manner. Knee flexion should not exceed 90° during the first 4–6 weeks after surgery. During these weeks the immobilizer is maintained when walking and as protection except during the rehabilitation exercises described below, with partial weight bearing. The crutches are removed progressively, but they are mandatory during the first 4 weeks, depending on whether the surgery involves concomitant interventions such as meniscal repair. After the first 4 weeks, the patient can first dispense with the crutch on the operated side and then, one to two weeks later, remove the second crutch on demand.

The rehabilitation process begins 10 days after surgery. Static work associated with electrostimulation is carried out from the beginning of recovery in order to strengthen the hamstrings and quadriceps. The electrostimulation consisted of biphasic, rectangular, and symmetrical electrical current with a working frequency of 50 Hz. The rest and work periods alternated every 10 s. Once the crutches have been removed, active work begins by performing concentric strengthening on the quadriceps in a closed kinetic chain. After 3 months, open kinetic chain strengthening exercises are introduced. Exercises to strengthen the hamstrings are concentric with progressive loads. All knee strengthening training is combined with gluteal muscle strength training and progressive proprioception work.

### 2.5. Isokinetic Muscle Strength

Isokinetic muscle strength was measured in both knees at 6 months after surgery using an isokinetic dynamometer (Biodex System 3, Biodex Medical Systems, Shirley, NY, USA). This time-point was chosen as it is coincident with the surgeon’s follow-up after which the patient’s return to sport is assessed. In addition, as previously indicated, it is a time-point where this factor begins to be evaluated [11,12,13,14,15]. The isokinetic dynamometer was calibrated before each evaluation. The isokinetic testing was applied at the knee joint in the CON/CON setting at 90°/s (6 repetitions) and at 240°/s (15 repetitions). The patient was placed in a seated position, with the backrest at 90°, and the hip and both knees at 90° flexion. The exercise was performed with a range of movement from 90° knee flexion, where the exercise started, to full extension at 0°.

### 2.6. Outcome Evaluation

Peak torque values (maximum isokinetic contraction force) were obtained from the dynamometer data and used as primary outcome measures. From these values, we also calculated the LSI (LSI = operated peak torque/non-operated peak torque × 100) as well as the strength ratios between hamstring (H) and quadriceps (Q) muscles (H/Q = hamstring peak torque/quadriceps peak torque). LSI values above 90% were indicative of adequate functional recovery [19]. H/Q ratios below 0.60 at 90°/s and below 0.70 at 240°/s were classified as indicators of unbalanced musculature with potential risk of injury [21].

### 2.7. Statistical Analyses

Statistical analysis was performed with SPSS 20.0 (SPSS, Chicago, IL, USA). Distribution of the samples was assessed by Shapiro–Wilk’s test. Data were considered statistically significant when *p* < 0.05. Demographic and clinical variables were calculated as mean and standard deviation. Comparisons were performed by Student’s *t* test. Multivariate regression was performed to analyze the influence on LSI and H/Q values (dependent variables) of the patients and surgical variables (independent) considered collectively, calculating coefficients (B), *p* value, odds ratios (ORs), and 95% CI.

## 3. Results

### 3.1. Demographics and Patient Characteristics

The study analyzed a total of 71 patients (Table 1). The median age was 27.0 years (CI 23.0–32.0), with 25 female patients (35.2%) and 25 patients who practice sports professionally (35.2%). The most frequently performed surgery was primary surgery (56 cases, 78.9%), surgery using autograft (58 cases, 81.7%), and intraarticular graft placement (43 cases, 60.6%). Around half (50.7%) of patients underwent concomitant meniscal repair.

### 3.2. Isokinetic Values at 90°/s

**Figure 1:**
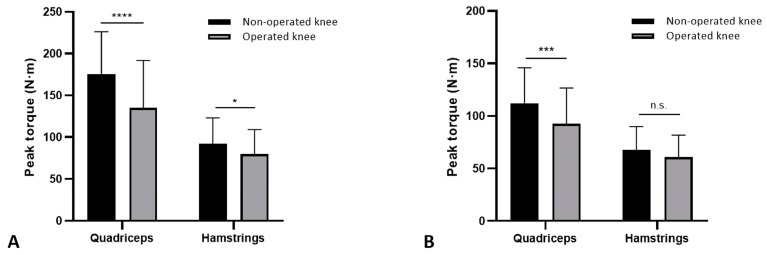
Peak torque values. Quadriceps and hamstring maximum strength values of the non-operated and the operated knee at speed of 90°/s (**A**) and speed of 240°/s (**B**). Error bars: SD; * *p* < 0.05; *** *p* < 0.001 **** *p* < 0.0001; n.s.: non-significant.

The peak torque was significantly lower at the operated knee for the quadriceps (135.2 ± 52.6 N-m vs. 175.6 ± 50.6 N-m; *p* < 0.0001) and the hamstrings (79.6 ± 29.4 N-m vs. 92.1 ± 31.2 N-m; *p* = 0.0157) (Figure 1A). The mean LSI value was 75.8 ± 17.2 with 57 cases (80.3%) having an LSI value below 90% for the quadriceps, and was 86.7 ± 13.2 with 43 patients (60.6%) showing LSI values lower than 90% for the hamstrings. The multivariate regression showed that younger age (*p* = 0.001) favored higher values of quadriceps LSI (Table 2) and meniscal repair (*p* = 0.037) for higher hamstrings LSI values (Table 3).

**Figure 2:**
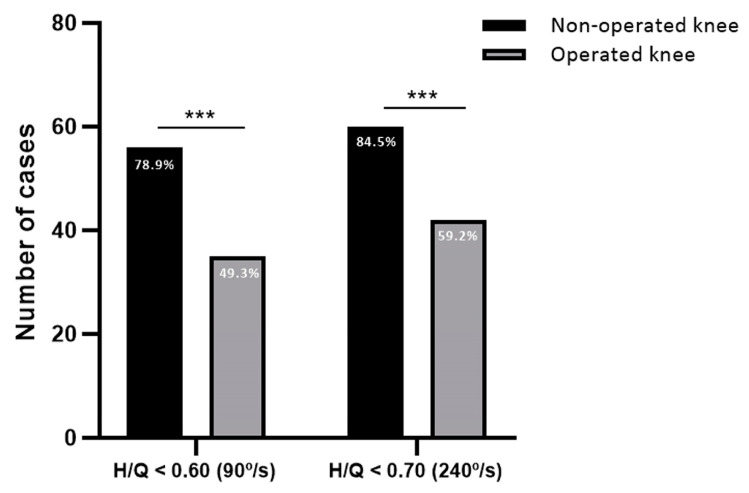
H/Q ratios of the non-operated and operated knee. Number of cases with H/Q ratio below 0.60 at speed of 90°/s and below 0.70 at speed of 240°/s. *** *p* < 0.001.

When analyzing the H/Q ratio, 56 cases (78.9%) showed a H/Q ratio below 0.60 in the non-operated knee which was significantly higher than the 35 cases in the operated knee (49.3%), with a difference of 29.6 points (CI: 13.9–43.4; *p* = 0.0002) (Figure 2). Patients with higher H/Q ratios were those of older age (*p* = 0.014), those undergoing revision surgery (*p* = 0.025) and those with autografts (*p* = 0.035) (Table 4).

### 3.3. Isokinetic Values at 240°/s

Quadriceps peak torque values were significantly lower at the operated limb for the quadriceps (92.6 N-m ± 34.1 vs. 112.1 N-m ± 33.8; *p* = 0.008) while no differences were found for the hamstrings (60.6 N-m ± 21.1 vs. 67.5 N-m ± 22.2; *p* > 0.05). The mean LSI value was 81.5 ± 15.3, with 49 cases (69.1%) having an LSI value below 90% for the quadriceps, and was 90.2 ± 12.1 with 37 cases (52.1%) showing LSI values lower than 90% for the hamstrings. Patients with higher LSI were those of younger age (*p* = 0.009) for the quadriceps LSI values (Table 5), while no variable significantly influenced the LSI values at the hamstrings (Table 6).

Regarding the H/Q ratio values, the number of cases in the non-operated knee below 0.70 was 60 (84.5%). In contrast, cases below 0.70 in the operated limb were 42 (59.2%), with a significant difference of 25.3 points less compared to the healthy knee (CI: 10.6–38.7; *p* = 0.0008) (Figure 2). The multivariate logistic regression model indicated that older age (*p* = 0.003) favored higher values of H/Q ratios (Table 7).

## 4. Discussion

The most important findings of this study were that there was a greater muscle unilateral imbalance in the non-operated knee than in the operated knee according to the H/Q ratio after 6 months of ACL reconstruction. The operated limb presented lower strength values than the non-operated knee, highlighted by not achieving 90% on LSI in most cases. Bilateral and unilateral balance on knee strength were conditioned by patient age and surgery-related factors. By associating factors derived from both the patient and the surgical procedure with quantifiable values associated with recovery, it would be possible to optimize patient management to improve restoration of muscle strength and identify those that may require further progressive resistance exercise [32].

The H/Q ratio relates hamstring and quadriceps muscle strength, being an indicator of unilateral muscle balance in the knee and therefore a possible indicator of future injury [33]. Although recent studies are cautious about using this indicator as a predictor of injury [29,34], the information it provides can be valuable for gaining insight into knee condition and return to activity [17,35,36,37]. Low H/Q ratios indicate weakened hamstrings compared to the quadriceps, which can be negative for the prevention of secondary ACL injuries due to the fact that hamstrings are involved in preventing an abrupt anterior tibial translation that negatively affects the ACL. A proper rehabilitation to restore the strength of hamstrings is critical to recovery after ACL reconstruction [30,38].

Surprisingly, our results showed greater knee unilateral imbalance in the non-operated limb than in the operated one 6 months after surgery, suggesting a higher risk of injury to the contralateral knee. After ACL surgery, the muscle strength of the non-operated knee can also decrease, and it is thus important to compare to the pre-injury isokinetic muscle strength (when available) or to normative data [15]. Recent studies have shown a high incidence of contralateral ACL injuries [10,39,40,41]. In a study with a follow-up of at least 10 years, Grassi et al. [42] found that the risk of second injury and revision surgery was two-fold higher for the contralateral knee than in the ipsilateral knee. A study conducted on professional ski racers also showed a higher percentage of second injuries in the non-operated ACL than in the operated ACL [43]. These findings were also confirmed in a recent meta-analysis that reviewed ACL reconstruction studies in athletes and found a higher rate of rupture in the contralateral knee than in the ipsilateral knee [23]. This tendency could indicate an imbalanced rehabilitation that is more focused on the operated knee than on the unaffected knee [44].

Age was a significant prognostic factor for muscle unilateral imbalance (H/Q ratio) in the operated knee, with the youngest patients presenting the worst values. These results are in line with previous studies, in which younger patients are more prone to failure after ACL reconstruction [23,42,43,45,46]. The higher risk in younger patients may be related to these patients returning to more physically demanding activity after surgery than in older patients. Measuring strength indicators such as the H/Q ratio during rehabilitation of young patients could help to more strictly adhere to and complete the recovery plan [47].

Data from the current study also suggest that surgical variables such as revision surgery or the use of allografts favor better H/Q ratios. Although these results may be specific to this study, they confirm previous studies showing that knee function is not adversely affected by revision surgery compared to primary surgery [48,49] or by the use of allografts compared to autografts [50,51]. However, the other surgical variable analyzed, anterolateral reconstruction, did not show any significant influence on the isokinetic values. This is in line with previous studies in which the addition of this technique did not enhance the isokinetic assessment, although it improved the percentage of graft ruptures [52]. In contrast, a recent study by Lee et al. [53] observed better strength data in patients undergoing ACL reconstruction combined with anterolateral reconstruction. However, that work performed the isokinetic assessment at different speeds than the present study and did not analyze the H/Q ratios, which hampers direct comparisons. Furthermore, neither Lee’s et al. [53] study nor similar studies did not analyze other patient- and surgery-related variables together, so further research is indeed needed in this area [54].

The knee muscle strength of the injured knee was lower than the unaffected knee, with a greater imbalance (LSI) in the quadriceps than in the hamstrings. This deficit was more pronounced in the test performed at lower speeds (90°/s), possibly due to the fact that at lower speeds, the force generated is greater, while the 240°/s is more useful to measure muscle power (generation of torque at high velocity). This reflected that most patients (80.3%) have not yet achieved an LSI of more than 90%, which is the cut-off at which recovery after ACL reconstruction is considered appropriate [20]. However, recent studies showed that this value is difficult to achieve after 6 months and a longer recovery period is necessary to avoid functional deficits [55,56,57,58,59,60].

Some authors suggest that the type of graft used in surgery may condition the time to reach optimal LSI values. Milutinović et al. [36] observed that in quadriceps strength there was no difference between the use of bone-patellar tendon-bone and hamstring tendon during ACL reconstruction. In contrast, the use of hamstring tendon did mean a greater strength deficit in the hamstring muscles [36]. However, other authors found that the use of bone-patellar tendon-bone resulted in a slower achievement of the strength criteria [61]. In the present work, in which hamstring tendon was used as a graft, the deficit found in the quadriceps was greater than in the hamstring tendon that could be related to arthrogenic muscle inhibition [62]. Therefore, it is reasonable to assume that longer rehabilitation periods will lead to greater muscle recovery and mitigate muscle imbalances.

Contrary to the H/Q ratio data, younger age favors better LSI values in quadriceps strength, thus increasing the deficit between the operated and non-operated limb in both high-speed and low-speed isokinetic assessments. The influence of age is consistent with previous studies, which observed that patients who recovered after ACL reconstruction according to LSI values were younger than those who did not recover [59,63]. This could be due to reduced adherence to application protocols or to increased muscle weakness at older ages [64].

This study has several limitations. First, the absence of pre-injury strength values makes it impossible to compare the isokinetic data to the original strength pre-injury, especially because the strength on both limbs deteriorates after ACL surgery. Second, functionality data collected through other methods, such as a hop test or return to sport records, could have provided more insight into the patient’s recovery. Third, all patients were recruited from the same center, which may introduce selection bias in the results and limit generalizability (external validity); however, the surgical interventions were performed by different surgeons using a standard procedure. Similarly, the rehabilitation process involved a team of physiotherapists who followed the same process with the patients. Finally, the insufficient number of patients in some subgroups makes it difficult to draw solid conclusions for some variables in the multivariate regression models.

The results of the present study highlight values and parameters obtained from muscle strength data using isokinetic assessment. Isokinetic assessment should take part during the rehabilitation and could help in achieving a more successful recovery and return to sports. Associating these data with variables related to surgical technique such as allograft or anterolateral reconstruction could help the surgeon optimize surgical protocols. The patient-related and type of injury (which structures were injured) can also influence the long-term patient-reported outcomes from these injuries [65]. Clinicians should pay attention not only to the operated knee but also to the non-operated knee as the knee strength deteriorates on both lower limbs after ACL reconstruction. Early detection of muscle imbalance could prevent later capsuloligamentous instability and important muscle deficits that need to be resolved during earlier stages of rehabilitation.

## 5. Conclusions

Isokinetic assessment 6 months after ACL reconstruction indicated greater unilateral (H/Q ratio) muscle imbalance in the non-operated knee than in the operated knee. Most patients did not achieve the adequate LSI values after 6 months of rehabilitation, suggesting strength deficits can persist even after 6 months of rehabilitation. Isokinetic muscle strength was influenced by patient-related variables such as age, as well as surgery-related variables.

## Figures and Tables

**Table 1:** Demographic and clinical characteristics.

Parameter	N/Median (CI)/N (%)
N	71
Age	27.0 (23.0 to 32.0)
Female	25 (35.2)
Professional sports practice	25 (35.2)
Surgery	
Primary	56 (78.9)
Revision	15 (21.1)
Graft origin	
Autograft	58 (81.7)
Allograft	13 (18.3)
Anterolateral reconstruction	28 (39.4)
Meniscal repair	36 (50.7)

**Table 2:** Multivariate regression analysis for quadriceps LSI at 6 months at speed of 90°/s.

Variable	B	*p* Value	CI	OR
Age	−0.096	*0.027* *	0.8 to 0.9	0.908
Sex *(male*/*female)*	0.690	*0.388*	0.4 to 9.6	1.994
Professional sports practice *(no*/*yes)*	0.127	*0.858*	0.2 to 4.5	1.135
Surgery *(primary*/*revision)*	0.710	*0.613*	0.1 to 31.8	2.034
Graft origin *(autograft*/*allograft)*	−1.003	*0.491*	0.0 to 6.4	0.367
Anterolateral reconstruction *(no*/*yes)*	0.932	*0.337*	0.4 to 17.0	2.539
Meniscal repair *(no*/*yes)*	−1.045	*0.151*	0.1 to 1.5	0.352

B: coefficient; CI: 95% confidence interval; OR: odds ratio; * *p* < 0.05.

**Table 3:** Multivariate regression analysis for hamstrings LSI at 6 months at speed of 90°/s.

Variable	B	*p* Value	CI	OR
Age	−0.003	*0.885*	0.9 to 1.1	0.997
Sex *(male*/*female)*	0.747	*0.228*	0.6 to 7.1	2.110
Professional sports practice *(no*/*yes)*	−0.017	*0.978*	0.3 to 3.3	0.983
Surgery *(primary*/*revision)*	0.866	*0.416*	0.3 to 19.2	2.378
Graft origin *(autograft*/*allograft)*	−0.517	*0.628*	0.1 to 4.8	0.597
Anterolateral reconstruction *(no*/*yes)*	1.398	*0.135*	0.7 to 11.8	2.909
Meniscal repair *(no*/*yes)*	−1.081	*0.056*	0.1 to 1.0	0.339

B: coefficient; CI: 95% confidence interval; OR: odds ratio.

**Table 4:** Multivariate regression analysis for H/Q in the operated knee at 6 months at speed of 90°/s.

Variable	B	*p* Value	CI	OR
Age	0.067	0.040 *	1.0 to 1.1	1.049
Sex *(male*/*female)*	−0.268	*0.667*	0.2 to 2.6	0.765
Professional sports practice *(no*/*yes)*	0.012	*0.984*	0.3 to 3.3	1.012
Surgery *(primary*/*revision)*	−3.249	0.033 *	0.0 to 0.8	0.068
Graft origin *(autograft*/*allograft)*	3.101	0.045 *	1.1 to 142.1	12.216
Anterolateral reconstruction *(no*/*yes)*	0.490	*0.536*	0.4 to 7.7	1.632
Meniscal repair *(no*/*yes)*	0.258	*0.666*	0.4 to 4.2	1.294

B: coefficient; CI: 95% confidence interval; OR: odds ratio; * *p* < 0.05.

**Table 5:** Multivariate regression analysis for quadriceps LSI at 6 months at speed of 240°/s.

Variable	B	*p* Value	CI	OR
Age	−0.111	*0.004* **	0.8 to 0.9	0.886
Sex *(male*/*female)*	1.155	*0.124*	0.7 to 13.8	3.174
Professional sports practice *(no*/*yes)*	−0.262	*0.684*	0.2 to 2.7	0.770
Surgery *(primary*/*revision)*	−2.220	*0.105*	0.0 to 1.6	0.109
Graft origin *(autograft*/*allograft)*	0.561	*0.679*	0.1 to 25.1	1.752
Anterolateral reconstruction *(no*/*yes)*	1.585	*0.074*	0.8 to 44.0	6.073
Meniscal repair *(no*/*yes)*	−0.041	*0.950*	0.2 to 3.5	0.960

B: coefficient; CI: 95% confidence interval; OR: odds ratio; ** *p* < 0.01.

**Table 6:** Multivariate regression analysis for hamstrings LSI at 6 months at speed of 240°/s.

Variable	B	*p* Value	CI	OR
Age	0.010	*0.670*	0.9 to 1.1	1.010
Sex *(male*/*female)*	1.027	*0.077*	0.9 to 8.7	2.794
Professional sports practice *(no*/*yes)*	−0.317	*0.582*	0.2 to 2.3	0.728
Surgery *(primary*/*revision)*	1.087	*0.239*	0.5 to 18.1	2.964
Graft origin *(autograft*/*allograft)*	−0.712	*0.439*	0.1 to 2.9	0.491
Anterolateral reconstruction *(no*/*yes)*	−0.227	*0.748*	0.2 to 3.2	0.797
Meniscal repair *(no*/*yes)*	−0.026	*0.962*	0.3 to 2.8	0.974

B: coefficient; CI: 95% confidence interval; OR: odds ratio.

**Table 7:** Multivariate regression analysis for H/Q in the operated knee at 6 months at speed of 240°/s.

Variable	B	*p* Value	CI	OR
Age	0.085	*0.003* **	1.0 to 1.2	1.088
Sex *(male*/*female)*	−0.903	*0.182*	0.1 to 1.5	0.405
Professional sports practice *(no*/*yes)*	−0.169	*0.796*	0.2 to 3.1	0.845
Surgery *(primary*/*revision)*	−0.596	*0.591*	0.1 to 4.8	0.551
Graft origin *(autograft*/*allograft)*	2.046	*0.072*	0.8 to 71.7	7.740
Anterolateral reconstruction *(no*/*yes)*	0.379	*0.645*	0.3 to 7.3	1.460
Meniscal repair *(no*/*yes)*	0.529	*0.393*	0.5 to 5.7	1.696

B: coefficient; CI: 95% confidence interval; OR: odds ratio; ** *p* < 0.01.

## Data Availability

The data presented in this study are available within the article. Additional inquiries may be sent to the corresponding authors.
